# Sexual self-efficacy and its related factors among married women of reproductive age

**DOI:** 10.4314/ahs.v21i4.39

**Published:** 2021-12

**Authors:** Rafat Assarzadeh, Zahra Bostani Khalesi, Fatemeh Jafarzadeh-Kenarsari

**Affiliations:** 1 Student Research Committee, Guilan University of Medical Sciences, Rasht, Iran; 2 Social Determinants of Health Research Center, Guilan University of Medical Sciences, Rasht, Iran

**Keywords:** Efficacy, sexual behavior, women

## Abstract

Sexual self-efficacy (SSE) has also been cited as an important factor for healthy and satisfying sex. The purpose of this study was to determine SSE and its related factors among married women of reproductive age.

The present study is a cross-sectional, descriptive-analytical study. The research samples were 588 married women of reproductive age. A cluster sampling method is used to select participants. Data collection instruments were the socio-demographic form and the Sexual Self-Efficacy Scale-Female Functioning (SSES-F). Data analysis, Friedman, Multiple Linear Regression was performed through SPSS software version 16.

The highest and lowest score was related to body acceptance (77.78) and communication (69.66), respectively. The results showed that age (B= 0.471, P<0.001), marital satisfaction (B= 0.11.3, P<0.001), life satisfaction (B= 3.5, P<0.03) and the economic-social welfare satisfaction were related to SSE. We've found that Women with a higher Education, Employment, higher income, and Younger husbands had the highest SSE score.

The components of age, marital satisfaction, life satisfaction, and economic status affect the SSE of married women of reproductive age. The results of this study can be useful in the design and implementation of sexual health promotion interventions.

## Introduction

The SSE is a predictor of sexual function and sexual quality of life 1. SSE is one's belief in his/her potency to have successful sex 2. Efficacy is derived from Albert Bandura's social cognitive theory, the famous psychologist, who has indicated that it refers to people's beliefs and judgments to perform duties and responsibilities 3. Bandoura believes that the result of a person's belief in his/her ability to do a skill is much stronger than the results from the surroundings 4. He assumes that one's belief in successful sexual intercourse affects his sexual function 5. Researchers and sex therapists believe that SSE, as an important variable, plays a fundamental role in desirable sexual function 6. On the other hand, SSE has a direct influence on Social and Mental performance necessary for sexual satisfaction 1,7,8.

Generally, people with low self- efficacy avoid sexual activity and have less sexual satisfaction 9. Indeed, higher SSE can improve sexual activities and sexual compatibility^7^. Also, Vaziri et al showed that SSE is associated with marital satisfaction. So that the SSE level is an important predictor of women's marital satisfaction 10. SSE can be influenced by other people, especially sexual partners 11. Melanie showed that a good relationship with a spouse and high personal autonomy in women is associated with increased SSE 12.

Since the sexual response and sexual function, is highly influenced by the self-efficacy of a woman, the ability of the women to maintain pleasurable sexual function depends on their self-efficacy and motivation to be willing and able to fulfill sexual activity 13. Thus, improving sexual self-efficacy can be a way to improve the sexual quality of life.

On the other hand, culturally, women's sexuality is framed as responsive and passive to male sexuality, with men initiating sex and women being the gatekeepers of sexual activity 14. This framing may undermine women's understanding of themselves as active participants in sexual activity 15. Improved self-efficacy can be associated with an increased ability of women to counteract this passive framing and advocate for their own pleasures and desires 16. Therefore, the concept of SSE in women needs to be clarified. Understanding self-efficacy along with the extensive determination of related factors in a sexual context can be contributing to a better understanding of women's sexuality and solving their sexual problems and empower women in sexual function 14. Additionally, little research has examined potentially related factors of SSE is expressed, as most SSE research focuses exclusively on adolescents. This study, therefore, aimed to identify the SSE and its related factors among married women of reproductive age.

## Methods

The present study is a cross-sectional, descriptive, and analytical study, which included 588 married women of reproductive age. The study population was selected based on some criteria. They were: women aged 15–49, married women who lived with their husbands, interested in participating, having the ability to read and write, and not using psychiatric drugs. The study population was selected using a Cluster Sampling method. At first, 24 Health Centers under the coverage of Guilan University of Medical Sciences were classified into 4 clusters based on geographic areas: North, South, East, and West. Then, using a random number table, 8 centers were selected by allocating 2 centers in each area.

Data were collected using a two questionnaire includes demographics, the standardized tool SSES-F. The demographic questions consisted of items regarding personal- social characteristics (age, education, career, alcohol and drug usage, smoking, marital and life satisfaction, economic status, having a child, number of children, youngest child age, infertility history, marriage history, history of psychiatric use), personal- social characteristics of the spouse (age, education, career, average monthly household income) were evaluated.

Bailes et al. 17 have developed SSES-F, and that has been confirmed by Rajabi et al. 18 in Iran. This questionnaire is a scale for the measurement of perceived ability in terms of emotional aspects, behavioral, and cognitive of women's sexual response and includes 28 items in a 5-point Likert type (sorted from 5=very high to 1=very low). The tool measures four cycles of women's sexual response: Interest, desire, arousal, and orgasm. Its sub-scales are included: Interpersonal desire, physical acceptance, communication Interpersonal, sensuality, orgasm and interest, personal arousal, refusal, and affection. Cronbach´s α coefficients were α=0.93 for the entire measure, with alphas ranging from 0.80 to 0.92 for individual factors.

The range of validity index was between 10 (absolutely uncertain) to 100 (quite sure) while the participants had been the ability to perform the sexual activity. If they are unable to perform, a zero score would be calculated. The overall grading of the questionnaire is computed from the mean scores of all aspects in SSES-F. The grading of each dimension is calculated based on the mean scores of the dimension. The highest score is 100 which represents a high SSE level.

The independent-samples t-test, Variance Analysis, and Pearson Correlation Coefficiencyis used to determine SSE based on socio-demographic variables of participants. Logistic Regression or Multiple Linear Regression was used to determine related factors to SSE. P-Value was considered with P <0.05.

## Results

The majority of participants (87%) were under 40 years old, diploma (47.1) and nearly 50% were unemployed (housewives). The majority's income was less than one million (about 50%). Besides, nearly 64% had children and about 15% had a history of infertility. The age of participants' spouses was 35. 72±7.61 (Table 1).

**Table 1:** Descriptive statistics for participants socio-demographic factors (N=588)

Variable		Numbers	Percentage
**Age**	< 30 years old	277	47.27
	30–39	247	42.15
	>40 years old	62	10.58
**Education level**	Illiterate	5	0.85
	Elementary	32	5.46
	3^rd^ grage junior	84	14.33
	Diploma	276	47.1
	University edu.	189	32.25
**Employment** **Status**	Unemployed	274	47.82
	Laborer	37	6.46
	Farmer	31	5.41
	Employee	170	29.67
	Self- employed	61	10.65
**Alcohol** **and Substance** **use**	Yes	47	8.15
	No	530	91.85
**Smoking**	Yes	73	12.46
	No	513	87.54
**Marital** **satisfaction**	Yes	467	79.69
	No	119	20.31
**Life satisfaction**	Yes	431	73.55
	No	155	26.45
**Economic** **satisfaction**	Yes	386	65.87
	No	200	43.13
**Monthly income**	Less than 1 million	270	49.72
	1–2 million	164	30.20
	>2 million	109	20.07

Based on the results, the total score of SSE (Mean score ± Standard Deviation) was 72.8±15 (Table 2). Maximum and Minimum scores are related to self- efficacy in body acceptance and communication, respectively.

**Table 2:** Distribution of SSE total and subscales

SSE score	Mean	Standard Deviation	Median	Confidence Interval 95% Mean		First Quartile	Third Quartile	Mean Rank	P-Value
				Low	High				
**Interpersonal** **orgasm**	69.98	16.97	71.43	68.6	71.36	60	82.86	4.15	< 0.001
**Interpersonal** **desire** **and interest**	76.07	16.32	75	74.74	77.39	65	90	5.83	
**Sensuality**	74.84	17.72	76.67	73.4	76.28	66.67	90	5.59	
**Personal** **arousal**	72.13	19.67	80	70.54	73.73	60	80	4.79	
**Affection**	72.39	18..08	70	70.92	73.86	60	80	4.78	
**Communication**	69.66	16.62	70	68.31	71.01	60	80	4.01	
**Physical** **acceptance**	78.77	16.7	80	76.43	79.14	70	90	6.03	
**Refusal**	71.06	24.85	80	69.04	73.07	60	80	5.04	
**Total SSE**	72.77	15	74.29	71.55	73.98	65.71	84.29	4.78	

In multiple analysis, multiple linear regression in the method of stepwise was used to determine predictors of SSE. In the initial model, all variables with a P <0.1 level of significance has joined the model. In the final model, 4 variables include age (B= 0.471, P<0.001), marital satisfaction (B= 0.11.3, P<0.001), life satisfaction (B= 2.6, P< 0.044) is SSE predictors. The efficacy score decreased with age. However, there is a significant relationship between marital, life, economic satisfaction with the SSE score (Table 3).

**Table 3:** Predictors of the women of reproductive age's SSE score using stepwise linear regression analysis (N=588)

Variable	Non-Standardized Coefficient		P-Value	Confidence Interval 95% Mean	
	Coefficient	Standard Deviation		High	Low
**Fixed Model**	108.622	3.179	<0.001	102.376	114.868
**Age**	-0.471	0.080	<0.001	-0.628	-3.14
**Marital Satisfaction**	11.30	1.586	<0.001	14.423	8.193
**Life Satisfaction**	3.446	1.580	<0.030	6.549	0.343
**Economic-** **social welfare, satisfaction**	2.622	1.296	>0.044	5.168	0.077

## Discussion

Based on the results, the total score of SSE in the participants was 72.8±15. Body acceptance had the maximum and communication had the minimum scores. A study by Zare et al (2015) that assessed the relationship between SSE and sexual life quality was reported the mean score of SSE 49.61±12.63. These researchers demonstrated that, sexual refusal had the highest mean on the dimensions of SSE which is in line with the present study in terms of the highest mean in the SSE area 19.

The SSE score is statistically significant based on all socio-demographic variables. Participants with younger husbands, higher education and employment, and high incomes had great SSE scores. Previous research supports this idea. 20, 21. The current study also showed that four variables: age, marital satisfaction, life satisfaction, and economic-social welfare satisfaction are SSE predictors. So that, when age level increased, the rate of SSE decreased. Moreover, the study supported that SSE has a direct effect on marital, life, and economic satisfaction 9, 10, 11. These findings are agreeing with the results of Vaziri et al. who noted SSE is associated with marital satisfaction 20. Different studies have indicated that sexual satisfaction is broadly related to marital satisfaction. On the other hand, the concept of marital sexual satisfaction, whether in the form of sexual activity or emotional satisfaction, involves one's self- efficacy cognition 22. Therefore, SSE has a significant contribution to marital satisfaction 23. Steinke et al. reported that marital satisfaction is associated with sexual desire. Indeed, there is a positive correlation between sexual satisfaction and marital satisfaction 10. Melanie found that SSE is enhanced among people who have high personal autonomy and good communication with their partners. As the findings showed, SSE is an important factor in creating a healthy and satisfying sexual relationship^12^. Azarian et al. also showed that women with a high level of SSE and the high quality of sexual life had more marital satisfaction 9.

## Conclusion

Often researchers are interested to evaluate women's sexual function, although that is an important one issue, understanding a women's sexual function is much more complex because influenced by women's sexuality. In order to advance the understanding of women's sexuality, a clarifying of some basic sexual concepts, and identifying the factors associated with them is essential and can be used in other areas of sexual research. As a sexual concept, SSE has great potential to help to understand women's sexuality and solves their sexual problems.

The highest and lowest scores of SSE were related to body acceptance and communication, respectively. Besides, age, marital satisfaction, life satisfaction, and economic-social welfare, satisfaction are the factors related to SSE. The results of this study can be important for the application to programs such as sexual education. Future study efforts should continue to include each gender and age group.
